# Trans-sectoral care in patients with colorectal cancer: Protocol of the randomized controlled multi-center trial Supportive Cancer Care Networkers (SCAN)

**DOI:** 10.1186/s12885-015-2002-6

**Published:** 2015-12-22

**Authors:** Alexander Bauer, Dirk Vordermark, Thomas Seufferlein, Hans-Joachim Schmoll, Henning Dralle, Wilfried Mau, Susanne Unverzagt, Stephanie Boese, Eva-Maria Fach, Margarete Landenberger

**Affiliations:** 1 Martin-Luther-University Halle-Wittenberg, Medical Faculty, Institute for Health and Nursing Science, Magdeburger Strasse 8, D-06112 Halle, Germany; 2 Department of Radiation Oncology, University Hospital Halle, Ernst-Grube-Strasse 40, D-06120 Halle, Germany; 3 Department of Internal Medicine I, University Hospital Ulm, Albert Einstein Allee 23, Ulm, D-89081 Germany; 4 University Hospital Halle, Clinic for Internal Medicine IV, Ernst-Grube-Strasse 40, D-06120 Halle, Germany; 5 Department of General, Visceral and Vascular Surgery, University Hospital, Ernst-Grube-Strasse 40, D-06120 Halle, Germany; 6 Martin-Luther-University Halle-Wittenberg, Medical Faculty, Institute for Rehabilitation Medicine, Magdeburger Strasse 8, D-06112 Halle, Germany; 7 Martin-Luther-University Halle-Wittenberg, Medical Faculty, Institute for Medical Epidemiology, Biostatistics and Informatics, Magdeburger Strasse 8, D-06112 Halle, Germany

**Keywords:** Colorectal cancer, Symptom management, Supportive needs, Care transition, Quality of life

## Abstract

**Background:**

Managing therapy-related side-effects and improving health-related quality of life in patients with colorectal cancer is still challenging. The need for an effective management of adverse events and unmet supportive care needs have been widely discussed. In the past decade, interventions by nursing staff gained more and more importance. Evidence suggests that a majority of patients even in early stages of the disease experience substantial impairments potentially resulting in diminished therapy adherence as well as impaired quality of life. However, evidence for the effectiveness of nurse-led interventions on symptom management and quality of life is still very limited. This especially applies to care transitions between different inpatient and outpatient health care providers throughout the course of treatment and aftercare.

**Methods/Design:**

Supportive Cancer Care Networkers (SCAN) is a prospective randomized controlled trial conducted in eight large and middle-sized German cancer centers and municipal hospitals. The target population is adults with colorectal cancer UICC I-III after initial R-0 resection scheduled for adjuvant chemotherapy or guideline-based aftercare only. 370 patients will be randomly assigned to either intervention or control group. Patients in the intervention group will receive an additional support by specialized oncology nurses for eight weeks after discharge from hospital by telephone, consisting of symptom monitoring, counselling on self-assessment and self-management and dealing with individual resources for coping and psychosocial well-being. The primary endpoint will be health-related quality of life (HRQoL) at eight weeks after discharge from the initial treating hospital.

**Discussion:**

The presented SCAN trial is to provide information that will be useful to advance our understanding of complex interdependencies between symptom severity, supportive care needs, functioning and the risk for diminished HRQoL. Most importantly, these patient-reported outcomes are not fully implemented in today’s clinical routine practice potentially resulting in therapy cessations and lower chemotherapy treatment rates for colorectal cancer especially in older patients.

**Trial registration:**

ClinicalTrials.gov Identifier NCT01651832.

## Background

### Incidence and prevalence

Colorectal cancer (CRC) is the second most frequent malignant disease in Germany with 62,430 newly diagnosed cases in 2010, 5-year prevalence of 214,300 and with more than 25,000 cases persistently the second most frequent cause of cancer death [1]. During the past decade, 5-year relative survival from colorectal cancer increased to 65 %. Despite of effective primary prevention, prevalence will continue to grow at over 3 % per year [2]. Thus, a growing number of survivors of colorectal cancer are likely to experience unmet supportive care needs (SCN) during the course of treatment to a different extent as well [3]. We are now questioning the possibilities of covering those supportive needs properly without exceeding personnel and financial resources.

### Unmet supportive needs

Recently published studies revealed a significant share of patients whose supportive needs are not covered entirely within routine care practice today [4]. Unmet supportive needs in turn have been proven to be significantly associated with diminished health-related quality of life (HRQoL). Such unmet supportive needs can refer to either physical or psychological symptoms as well as accessibility of health care or social welfare benefits. Meeting these needs is likely to improve the patients’ quality of life by lowering symptom severity and psychological distress and might prevent dissatisfaction with health care [5–8].

But, while the assessment of cancer or therapy-related symptoms is more and more integrated into routine supportive therapy, the assessment of SCN still lacks a systematic implementation [9–11]. An especially critical phase in the course of treatment for not addressing the patients’ supportive needs sufficiently is the transition from in-patient primary therapy to out-patient aftercare [12]. Especially in more than 30 % of elderly patients (65+) chemotherapies are discontinued within 1–4 months due to persistently high levels of symptoms and functional impairments, leading to significantly diminished survival [13]. Therefore, national and international guidelines on the treatment of colorectal cancer recommend optimizing treatment of side-effects and cancer-related symptoms seamlessly [14–16]. Hence, a systematic monitoring is necessary for the treatment of symptoms and impairments [16].

Recent reference data from the QUASAR2-trial [17] proved that up to 30 % of patients suffer from substantial therapy-induced symptoms during active therapy. Depending on the regime, common toxicities in patients with colorectal cancer are diarrhea, nausea and vomiting, mucositis/stomatitis, myelosuppression, and hand-foot syndrome. This may lead to avoidable impairments in HRQoL as it is often found in patients with colorectal cancer [18–20]. Moreover, the necessity for follow-up treatment can last for years after initial diagnosis and treatment as a recent analysis in long-term survivors in the US with leukemia, bladder, and colorectal cancer showed [21]. And even without being indicated to adjuvant chemotherapy, patients in early tumor stages usually state increased psychosocial and medical supportive needs, especially after surgery [22].

### Nursing interventions to address SCN

In the past, nursing interventions for the improvement of access to the care for cancer patients, for the support in managing therapy-related side-effects, for the improvement of therapy compliance and care continuity [23] as well as for the improved treatment management have gained importance [10, 24–26]. The early detection of therapy-related symptoms and functional restrictions by nursing staff can contribute particularly to the improvement of outpatient aftercare and sustain patients’ motivation for therapy compliance [17]. Additional information on patients’ medical condition using patient-reported outcomes (PROs) can contribute to support professionals involved in clinical decision making [25, 27].

Problem for cancer and other chronic diseases is the fact that the medical condition as well as the resulting supportive needs change during the course of the disease and are hardly predictable [10]. That is why the routine collection of relevant disease parameters and supportive care need measures within integrated care concepts is essential during the entire course of the treatment. We were able to show that due to nursing interventions symptom-related supportive care needs concerning disease- or treatment-related side-effects (nausea, vomiting, pain, fatigue, malnutrition) can partially be addressed beyond inpatient treatment by conveying self-management abilities and an attitude change in patients regarding cancer [28–30].

In general, there is still a lack of nursing interventions that cover unmet SCN of all different aspects during primary therapy and care transition. Random models have been evaluated within clinical trials, such as e.g. patient navigators, either based on nursing staff or lay persons. Recently, special attention was paid e.g., on such guidance throughout the course of treatment in a study by Wagner et al. [31]. The *Nurse Navigator* program providing additional support regarding care access, distress and fatigue assessment by telephone for 4 months was compared to usual care. While patients in the *Nurse Navigator* group stated improved experience and reduced problems in care access, the overall HRQoL was not affected significantly. These findings might not be standing in contrast to prior findings. As a study on the newly developed Comprehensive Concerns Assessment Tool and the European Organization for Research and Treatment of Cancer (EORTC) showed, the most important factors were related to self-management abilities, psychological symptoms and medical information rather than the usual suspects, i.e. pain or gastrointestinal symptoms [32]. Furthermore, continuous access to qualified health care providers and other resources for the management of long-term debilitating symptoms is essential from the patient’s view even for years after the initial diagnosis and treatment [33]. There is preliminary evidence for the feasibility and acceptance of an electronic monitoring of symptoms that could be useful for clinical decision making, too [34]. Thus, it is to be assumed that prior standardized interventions as mentioned above failed to address the entire variety of changing unmet SCN in differing phases of the care process sufficiently, leading to a possible mismatch between the patient’s current condition and the support provided. Following the state of research, in these subjects a personalized approach as proposed in this paper, can take on an important role. Moreover, our approach is likely to add in-depth information on interdependencies between different domains of SCN in order to facilitate the promotion of HRQoL effectively.

### Objectives

The main purpose of this study is to increase the proportion of patients achieving a clinical relevant improvement of their health-related quality of life of 10pts. [35] by 15 % compared to usual care. Secondary objectives are to

1. Determine the prevalence, time of occurrence and severity of unmet SCN,
2. Analyze effective elements of nurse-led patient counselling on patient self-management, self-assessment and knowledge,
3. Identify individual resources that medical professionals can strengthen and utilize to build up the patient’s autonomy and coping abilities and
4. Determine the effect of a supportive nurse-led intervention on disease-free survival 8 months after surgery.

## Methods/Design

### Study design

SCAN is a prospective randomized controlled multi-center trial in adult patients with colorectal cancer (International Classification of Diseases, ICD-10 C18-20) in Union internationale contre le cancer (UICC) stage I-III. The study is conducted in the surgical wards of eight large and middle-sized cancer centers and general hospitals in Saxony-Anhalt and Saxony, Germany.

Patients randomly assigned to the intervention arm receive additional support by specially trained oncology nurses for eight weeks after discharge from hospital following surgical therapy. Allocation to either intervention or control group is performed with equal ratio. The following amendments were submitted to the local ethics committee and approved on June 4^th^ 2013:

1. Expansion of eligibility criteria to UICC stage I and
2. Change of the primary outcome from adjuvant chemotherapy utilization to HRQoL.

Both amendments are causally linked to one another. Prior to the amendment only patients with UICC stage II & III were eligible for trial inclusion because of the actual medical guideline for the treatment of colorectal cancer. According to this guideline, adjuvant chemotherapy is indicated in patients with UICC stage II and more advanced tumor states only. Due to upcoming effects of the establishment of the preventive colonoscopy as a standard benefit of the German statutory health insurance a noticeable shift regarding tumor stages is obvious. Studies, which were published after approval of the study by the sponsor show that colorectal cancer, despite of the low participation rate of the healthy general population is detected significantly more often in early stages (UICC I) [36, 37] than previous reference data suggested. Nevertheless, this positive effect leads to a significant reduction of the patients eligible for the study. With the additional inclusion of patients in the UICC stage I a rise of the case number of approx. 40 % could be achieved. At the same time, patients in UICC I are not indicated to adjuvant chemotherapy. As a result, widening the eligibility criteria had to be accompanied by changing the primary outcome. Since HRQoL had been defined as a central secondary outcome, the primary outcome was replaced by a former secondary one without changing elements or procedures of the intervention itself.

### Study population

The target population is adult patients with colorectal cancer (International Classification of Diseases, ICD-10 C18-C20, UICC I-III) in curative therapy conditions. Eligible patients will be identified after surgery by hospital physicians through histological and pathological findings. Eligible patients expressing interest in taking part in the study will be contacted by a specially trained oncology nurse and an initial appointment will be scheduled. Fully informed written consent will be obtained before collecting any data or providing counseling.

### Inclusion criteria

- Being treated surgically at one of the participating study centers for colorectal cancer (R0)
- Age 18–85 years.
- ECOG performance status 0–2
- Prospective further life expectancy of >3 months.
- Living in Saxony-Anhalt or Saxony.
- Ability to participate in regular follow-up intervals as determined in the medical guideline
- Informed written consent must be obtained according to ICH/EU GCP, before trial inclusion.

### Exclusion criteria

- Contraindications for surgical therapy, according to the medical guideline, e.g. Inadequate liver, bone marrow, and kidney function or coronary heart disease (NYHA III-IV).
- Lacking ability to understand, speak and write German
- Being admitted to a nursing home permanently.

### Primary endpoint

The effectiveness of SCAN program will be assessed on an individual patient level by comparing the proportion of patients who achieved a clinical relevant improvement of their HRQoL by 10 pts. At 8 weeks after discharge from hospital.

### Secondary endpoints

An overview on primary and secondary endpoints and the study timeline is provided in Table 1:

**Table 1:** Outcome measures and measure times

Measure times		T0	T1	T2
Outcomes & Instruments		Baseline (day before discharge)	Primary measurement (8 weeks after discharge)	Follow-up (8 months after discharge)
HRQoL (primary outcome)	EORTC QLQ-C30 + EORTC-QLQ CR29	x	x	x
Utilization of adjuvant chemotherapy			x (if appropriate)	x
Supportive Care Needs and individual resources	FU-T 37		x	x
Distress	Distress Thermometer (DT)		x	x
Symptoms and functional impairments	MD Anderson Symptom Inventory		x	x

### Sample size

For sample size calculation the portion of the patients who reach a clinically relevant improvement of the global health-related quality of life (HRQoL) within 8 weeks after discharge from hospital is chosen. The sample size calculation is based on an expected response difference of 15% points in the subscale *“global health status”*/QoL of QLQ-C30 EORTC between the intervention and the standard treatment 8 weeks after the clinic dismissal after surgical therapy. Own reference data [30] show a response of 48 % in standard treatment, so that in the intervention group responses of 63 % are expected. To be able to detect a relevant effect of the intervention with a rise of the responses by 15% points with 80 % power, *n* = 370 patients (*n* = 185 patients per study arm) are needed. Including a dropout rate of approx. 10 %, *n* = 406 patients (*n* = 203 per study arm) will be recruited.

### Randomization

Randomization on an individual level, patient enrollment and group allocation will be performed by fax from the Institute of Medical Epidemiology, Biostatistics, and Informatics (IMEBI), Halle as an external and independent unit. Randomization will be stratified by center using blocks of variable length from a reproducible SAS PROC PLAN code. A consecutive analysis of non-responders and of the causes for changes in therapy regimes is intended additionally due to the high therapy-related impairments in wellbeing and quality of life during adjuvant chemotherapy.

### Blinding

Patients are blinded for this study. A distinct patient number is allocated in case of inclusion in the study. A blinding of the medical staff (physicians, oncology nurses) is impossible due to the frequent re-admission of patients to be expected and the disclosure of information in the patient-held records.

### Study procedures

**Fig. 1:**
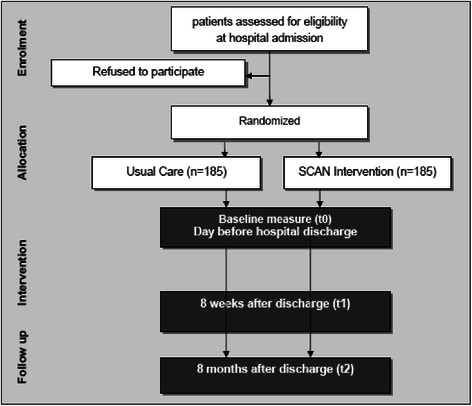
Study flowchart/CONSORT diagram

**Fig. 2:**
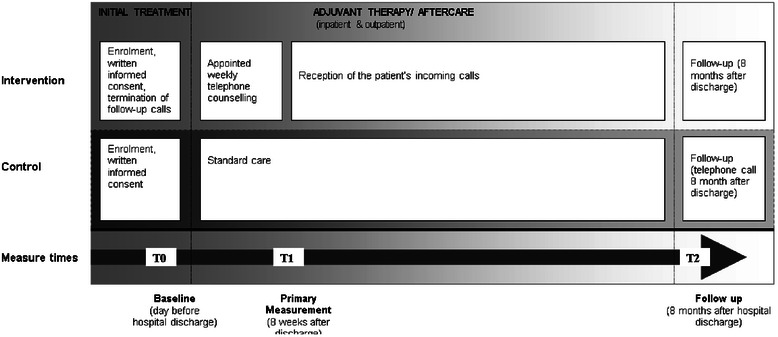
Time line and procedures

The study flowchart is presented in Fig. 1. The study timeline is presented in Fig. 2.

### Intervention

Feasible patients are assessed by the participating study centers and registered to the coordinating trial center. Patients meeting the inclusion criteria and signing a written informed consent are randomized into the study arms. Patients in the intervention group are offered an additional nursing intervention after discharge for 8 weeks. The intervention time-line was adjusted to the actual S3-guideline on the treatment of colorectal cancer regarding the recommended latest onset of adjuvant chemotherapy. The intervention consists of a standardized protocol-based telephone follow-up serving once per week to disclose patients’ current supportive needs and in order to detect therapy-related physical and psychological impairments as early as possible. Standardized protocols were developed beforehand to ensure both consistent assessment and patient information during the counseling. Additionally, the SCAN is at patients’ disposal for contact from the beginning of primary therapy during the inpatient stage to outpatient aftercare for up to eight months. The intervention covers the entire period of transition from acute clinical care to out-patient aftercare.

### Training of the nursing staff

Incidents classified as critical were processed beforehand by the research group into a telephone guideline. These incidents and appropriate counseling facilities are included in the training program prior to the implementation of the intervention. Specialist oncology nurses designated to carry out the intervention underwent a two-day training (a total of 18 h) conducted by clinical oncologists and members of the study group.

### In-patient period

Patients in the intervention group are visited twice by a specially trained oncology nurse (Supportive Cancer Care Networker, SCAN) during their inpatient stay. Both visits take about 30 to 60 min. Fist, patients are given relevant information, i.e. contact with specialists, voluntary services and the next steps and appointments of the treatment plan on the second last day before discharge from hospital. Second, at the last day before discharge the SCAN takes up the contact information and appoints weekly telephone consultations for eight weeks. Patients are handed out written information materials and the study documents, as for example, patient-held records (PHR), are explained. The PHR is derived from a newly generated 37-item generic supportive needs questionnaire “*Fragebogen zur Erfassung von Unterstützungsbedarfen von Tumorpatienten*“(FU-T). To ensure that patients are enabled to inform structured and completely about relevant changes of their medical condition, filling in the PHR is practiced under instructions. In coordination with the treating clinicians, critical events in which an immediate contact to the SCAN should occur are listed individually.

### Out-patient period

During the outpatient intervention period the SCAN inquires therapy-related symptoms and functional limitations of patients regularly using patient-held records (PHR) in order to assist patients during the transition from inpatient acute care to outpatient aftercare. In the first eight weeks after hospital discharge obligatory telephone consultations are carried out by the SCAN. During the consultations the SCAN rates the urgency of unmet supportive needs, incorporates qualified help if necessary and supports communication of involved health care professionals. Support is provided in five categories of possible supportive needs following standardized protocols:

1. Proper self-management strategies for physical and psychological symptoms
  Patients stating persistent physical or psychological symptoms are guided in effective self-assessment possibilities. Moreover, depending on the kind of symptom suitable self-management strategies are discussed by the SCAN.
2. Warranty of care continuity
  The entries in the PHR are discussed on the phone with a SCAN regarding critical incidents. Here, the individually determined critical events and processes are forwarded to attending hospitals or resident physicians in order to initiate treatments appropriate to the occurring problems and to call in or hospitalize patients. Additionally, patients have the possibility to call between telephone appointments in case of therapy-related complications and side-effects and of psycho-oncological or logistic problems. An early detection of therapy-related physical and psychological impairments also aims at optimizing treatment management by providing an almost real time feedback to the medical professionals.
3. Informed decision making
  Beginning at the in-patient consultations, detailed information on the therapy plan as well as alternatives is provided. Further information needs are addressed during the appointed telephone calls, depending on the occurrence of events in need for support, e.g. pharmacological and non-pharmacological supportive measures, or referred to qualified external health care professionals and self-help groups.
4. Participation
  In addition, within the entire out-patient intervention period, patients are guided in access to office-based oncology specialists and organizational or logistical barriers to medical treatment facilities are discussed with the SCAN. Furthermore, patients are actively encouraged to keep a high therapy adherence, especially in patients living in rural areas or the socioeconomically deprived.
5. Psycho-social well-being
  The consultation is aimed individually on the patient. Counseling on psychological well-being pursues an improvement of the quality of life by the support of the psychic as well as the social functionality of the patient, which is possibly a prognostic factor for the overall survival [10, 27]. Thereby, special attention is paid to individual resources of the patients. Patients are aided in defining both useful individual and institutional resources, e.g. experiences of self-efficacy or available social security benefits.

### Follow-up

In order to determine differences in healthcare services utilization between intervention group and controls, all patients are asked to fill out additional written questionnaires at 8 weeks and 8 months after discharge from hospital. At both times, patient’s adherence to medical recommendations and completion of medical therapies is assessed as well.

### Potential adverse events

The SCAN intervention with a non-invasive educational profile is considered to include no additional risk.

### Removal from the study

Subjects may refuse to participate in the study at any time without giving a reason. The following further reasons can lead to withdrawal of the study for single patients:

- Wish of the patient,
- non-compliance,
- Not the predictable health disturbances which force the patient to the withdrawal, according to the appraisal of the treating doctor,
- Death of the patient.

### Statistics

#### Primary endpoint analysis

The analysis of the primary outcome will obey to the intention-to-treat principle using generalized linear regression modelling and reported as relative risk with its according 95 % confidence interval. The model will be adjusted for cancer site, tumor stage, cancer therapy, ECOG performance status, age, sex and eventual previous neoadjuvant radiation therapies.

#### Secondary endpoint analysis

Secondary endpoints will be assessed by the respective two-group tests with a corresponding 95 % confidence interval. For each secondary endpoint specific statistical tests will be conducted. That is, for continuous outcomes the *t*-test will be used, for binary outcomes the chi-square-test and for time-to event outcomes the log-rank test. Secondary endpoint analyses are considered as exploratory. Subgroup analyses will be undertaken by checking interaction of covariates with intervention [38]. The trial will reported according to the guidelines of the consolidated standards of reporting trials (CONSORT) for randomized controlled trials. Adverse events will be reported as rates with corresponding 95 % confidence intervals. All adverse events (AEs) and serious adverse events (SAEs) will be given in lists.

### Supplementary qualitative analysis

Due to the large amount of free text data gathered in the PHR, a supplementary qualitative analysis based on the approach by Maying [39] will be carried out. It will be based on a specific category system underlying the incorporated FU-T questionnaire. Developing the final categories therefore is performed inductively from the material on the free text data by summary, explication and structuring. The categories are defined and collected together with demarcation rules and anchor examples in a code-decode guide which forms the guideline for the classification of passages. Based on this code-decode guide the free text data are explored and re-categorized if necessary. After the final production of category system and code-decode guide the content will be rehashed descriptively. These results are processed in graphical representations.

### Auditing

To realize quality assurance the Institute for Health and Nursing Science as the coordinating center of the study will provide an advanced support for documentation and study management in the recruiting centers. Trained research staff will visit each center at least 4 times. Pre-study-visit (1): the practice team will be introduced into the trial procedures, qualification of practice staff and research capacities building and documentation will be checked. Study visit (2 & 3): support for follow-up and data management with special attention on reporting AEs and SAEs. Post-study-visit (4): final query management. Additional audits can occur at any time during or after completion of the study. Monitoring will be performed regularly by verifying key data (signed informed consent form, inclusion and exclusion criteria and key baseline data). The research staff will be trained in source data verification and will be supported in preparing standardized reports.

To ensure the implementation of the intervention, quality audits are being conducted in the intervention groups in all study centers based on the guidelines of the Royal Collage of Nursing [40] and the German Network of Quality Assurance in Nursing Care [41]. The audit is based on (1) regular monitoring of the trained nurses’ knowledge about the training module, and (2) on an additional comparison of nursing records with study documentation for 10 % of all included patients. Nurses who administer the interventions and assess the outcomes are aware of group allocation due to their participation in the training to manage the SCAN intervention. Patients are not informed about group assignment, but might be aware of it due to unmasking information from nurses. Group allocation perception of included patients will be assessed at follow-up.

### Ethical matters

The study is performed according to the ICH-GCP principles and is approved by the local ethics committee of the Medical Faculty at the Martin-Luther-University and regional ethics committees of the participating centers. The trial is registered in the clinical trial registry ClinicalTrials.gov.

## Discussion

During the past two decades, nurse-led telephone-based care coordination programs to improve outcomes such as HRQoL after surgical resection for colorectal cancer have gained increasing importance. However, nurse-led case management concentrating on information provision and care pathway supervision alone was not effective in promoting HRQoL [19]. As reported by Gray et al. [42], there are both modifiable and fixed factors predicting HRQoL. Especially an effective management of symptoms, e.g. anorexia, dyspnea, fatigue, depression and impaired activities, appears to be potential for interventions to improve HRQoL in patients with colorectal cancer. Recently, a qualitative analysis by Sun et al. [33] emphasized the importance of a long-term coordination of care in order to manage also persistent late-effects even for years after initial diagnosis. To this issue, newer research proved that an assessment of those symptoms is feasible and safe even in real-time reporting systems carried out via cell phone applications [43]. However, the available evidence is still very limited. A systematic review of controlled trials concluded that there is no clear evidence to date that unmet needs are indeed modifiable due to methodological issues and low statistical power or inadequate measures [44].

The Australian CONNECT study, e.g., with a comparable approach found no differences in unplanned readmission (25.6 % of interventions vs. 27.9 % of controls; *P* = 0.5). In this study also no significant differences in experience of care coordination, distress, or HRQoL between groups were achieved [45]. The authors concluded that the intervention was probably not intensive enough to be effective. Furthermore, the sample studies showed low levels of SCN in general which stands in contrast to prior findings from other studies in patients with colorectal cancer [46].

In contrast to these findings, a Chinese study comparing a self-efficacy enhancing intervention to standard care found a coincident significant improvement in their self-efficacy (F = 7.26, *p* = 0.003) with a reduction of symptom severity (F = 5.30, *p* = 0.01), symptom interference (F = 4.06, *p* = 0.025), anxiety (F = 6.04, *p* = 0.006) and depression (F = 6.96, *p* = 0.003) at three and six months [47]. However, no significant improvements in HRQoL were observed. In addition, a longitudinal prospective evaluation of toxicity conducted in the UK suggested that nurse-led telephone follow-up can potentially lead to reduced symptom severity (chest pain, vomiting, oral mucositis, nausea, insomnia) and has a similar impact on the management of some symptoms when compared to home care [48].

The findings of the SCAN trial will facilitate the prospective selection of individually appropriate strategies in the trans-sectoral management of patients with CRC and evaluate the effectiveness daily clinical routine.

The proposed SCAN trial, although targeting a similar population as the trials mentioned above, is different in several important aspects. First, we propose a more comprehensive screening of occurring SCN throughout the course of treatment. A previous trial [49] reported that toxicity management was most effective within the first two cycles of treatment. Afterwards, many symptoms are likely to reach a stable plateau. Taking these findings into account for the SCAN intervention, the critical time to provide supportive care interventions for symptom management and HRQoL enhancement is not only the first two weeks of chemotherapy, but also continuously during routine aftercare in order to avoid prolonged high levels of symptoms and functional impairments.

Second, the intensity of the study intervention is not limited to a specific timeline only, but also to the patient’s needs for additional support. As Reese et al. [50] recently showed, HRQoL, supportive needs as well as functioning are not only interdependent but also resulted in highly distinctive perceived illness burden. Thus, the individualized real-time SCAN intervention has the potential to meet the demands of subgroups of CRC patients more targeted regarding supportive care coordination and with it to alleviate upcoming illness burden at an early stage.

Third, there is an ongoing debate on how to optimize multi-professional care. An unsolved problem in routine care settings today with potentially high relevance for the proposed SCAN trial is the knowledge of the nursing staff. Especially, insufficient knowledge of cancer survivor issues [51] and also a lack of feasible measures has been shown to be an important barrier to providing appropriate follow-up care [52]. Besides, nurses’ recognition may not accurately reflect the patients’ supportive care needs in every aspect [53]. To eliminate this bias, we offer an extensive training of the physician and nursing staff prior to implementation of the SCAN intervention. This training will be incorporated continuously throughout the trial conduction.

In summary, data from this study will contribute to understanding the link between unmet SCN and the risk for diminished QoL by providing a more in-depth look into the individual perception of the differing importance of health and care-related problems occurring over time.
